# Early Neuroschistosomiasis Complicating Katayama Syndrome

**DOI:** 10.3201/eid1209.060113

**Published:** 2006-09

**Authors:** Jan Clerinx, Alfons van Gompel, Lut Lynen, Berten Ceulemans

**Affiliations:** * Institute of Tropical Medicine, Antwerp, Belgium;; † University Hospital Antwerp, Edegem, Belgium

**Keywords:** neuroschistosomiasis, travelers, Katayama syndrome, Schistosoma hematobium, letter

**To the Editor:** Neurologic complications of schistosomiasis may occur early as well as late in the course of infection; they result when a pair of worms becomes lodged in the vasculature, and their eggs become trapped in the microcirculation of the brain or spinal cord. There, they elicit a strong inflammatory response, which causes the clinical manifestations (*1**–**3*). Magnetic resonance and computed tomographic images of the brain show nonspecific, contrast-enhancing infiltrates, which suggests brain tumors (*4*). Definitive diagnosis requires finding *Schistosoma* eggs in feces, urine, rectal biopsy specimen, or biopsy specimen of central nervous system lesions (*5*), while a positive antibody test result provides a probable diagnosis only. To prevent irreversible damage, early treatment with corticosteroids is essential, after which the adult worms can be eliminated with praziquantel (*3**,**4*). A high degree of suspicion is therefore needed to avoid treatment delay.

Neuroschistosomiasis has been reported in persons living near Lake Malawi, in Malawi (*6*). Four members of a Belgian expatriate family (both parents and 2 children, a 12-year-old boy and a 7-year-old girl) went swimming in Lake Malawi in September 1998. On the advice of a physician, they took praziquantel 2 weeks afterward as postexposure prophylaxis. Nevertheless, fever, hypereosinophilia, cough, and abdominal discomfort developed in the mother and both children 6–8 weeks after they had been swimming; these symptoms were indicative of Katayama syndrome. The father remained asymptomatic but had a moderately raised eosinophil count (760 cells/mm^3^) and tested positive for *Schistosoma* antibodies. *Schistosoma hematobium* eggs were found in feces and urine of the mother and girl. All family members tested negative for schistosomiasis on a screening visit the previous year. The boy was admitted to a Zambian hospital because of high fever, cough, and a pulmonary infiltrate. He did not improve on antimicrobial drugs given for suspected pneumonia, and a gradually worsening neurologic syndrome developed, with left-sided hemiparesis, slurred speech, and slow movements.

The boy's condition prompted repatriation ≈10 weeks after the exposure. On admission at the University Hospital of Antwerp, his symptoms included fever, left-sided paresis with left-sided Babinski sign, and high eosinophil count (3,080 cells/mm^3^). An ELISA for *Schistosoma* antibodies was weakly positive. Examination of spinal fluid showed normal cell and protein content and a slightly lowered glycorrachia. A nuclear magnetic resonance (NMR) image of the brain showed multiple, small, contrast-enhanced white matter lesions around the semiovale center (cranially from the lateral ventricles) bilaterally and in the right parietal cortex. A tentative diagnosis of acute neuroschistosomiasis was made, and the patient was given corticosteroids with praziquantel, 750 mg twice a day for 14 days. At the end of this treatment, his condition had markedly improved; discrete hemiparesis was the only residual symptom. One month later, the patient had returned to normal, apart from left leg hyperreflexia. An NMR of the brain still showed residual lesions around the semiovale centers. Ten months later, results of clinical and neurologic examinations were normal, but NMR of the brain still showed minor residual lesions around the semiovale center on the right side. During follow-up, a serologic shift (indirect hemagglutination schistosomal antibody test) was seen, and eosinophil count decreased gradually to normal (Table). Although the boy never excreted eggs, *S. hematobium* infection was presumptively diagnosed on the basis of active infection in his relatives and the response to treatment.

**Table:** Evolution of acute neuroschistosomiasis in 12-year-old boy after treatment

Parameter	Day 0	Day 45	Month 10
Neurologic symptoms	Present	Present but diminished	Absent
Eosinophil count (per mm^3^)	3,080	1,030	370
*Schistosoma* ELISA	Weakly positive	Weakly positive	Negative
*Schistosoma* indirect hemagglutination assay (antibody titer)	Negative	640	80
Urine microscopic analysis	Normal	Not available	Normal
Urine concentration test for schistosomal eggs	Not available	Not available	Negative
Feces concentration test for schistosomal eggs	Not available	Not available	Negative

When neurologic symptoms appear soon after primary infection with *Schistosoma* flukes, confirming the diagnosis may prove difficult, and schistosomiasis should be suspected when the patient has bathed in potentially infected water. Furthermore, hypereosinophilia is an early warning sign, as seroconversion and egg excretion may be slower to evolve. Both elements provide sufficient circumstantial evidence to strongly suspect the diagnosis (*2*). In this case, the full-blown Katayama syndrome contributed to the evidence.

Praziquantel only kills adult worms and does not inactivate schistosomules, nor the miracidium inside the eggs, which will continue to elicit a damaging immunologic response for some time. Early antischistosomal treatment might, in fact, worsen symptoms (*7*). Because schistosomules may require up to 8 weeks to mature, early postexposure treatment with praziquantel cannot be used to forestall disease after primary infection. Furthermore, Katayama syndrome may occur as early as 3 weeks after exposure. On the other hand, withholding praziquantel until larvae have matured (8 weeks after exposure) would not prevent Katayama syndrome in many cases (*7*). Acute symptoms, including early neuroschistosomiasis, may therefore still develop during this 5-week window after exposure, despite early praziquantel administration.

Artemether has shown promising activity against schistosomules (*8*). Repeated administration throughout the transmission season has prevented Katayama syndrome in *S. japonicum* infection (*9*). Its use, singly or in combination with praziquantel, should be investigated as true postexposure prophylaxis for primary schistosomal infection in nonimmune travelers (*10*).
